# Numerical optimization and performance evaluation of ZnPC:PC70BM based dye-sensitized solar cell

**DOI:** 10.1038/s41598-023-37486-2

**Published:** 2023-06-27

**Authors:** Ghazi Aman Nowsherwan, Muhammad Aamir Iqbal, Sajid Ur Rehman, Aurang Zaib, Muhammad Irfan Sadiq, Muhammad Ammar Dogar, Muhammad Azhar, Siti Sarah Maidin, Syed Sajjad Hussain, Kareem Morsy, Jeong Ryeol Choi

**Affiliations:** 1 Centre of Excellence in Solid State Physics, University of the Punjab, Lahore, 54590 Pakistan; 2 School of Materials Science and Engineering, Zhejiang University, Hangzhou, 310027 China; 3 Central South University Changsha, Hunan, 410017 China; 4 Faculty of Data Science and Information Technology, INTI International University, 71800 Nilai, Malaysia; 5 Biology Department, College of Science, King Khalid University, Abha, 61421 Saudi Arabia; 6 School of Electronic Engineering, Kyonggi University, Suwon, Gyeonggi-do 16227 Republic of Korea

**Keywords:** Energy science and technology, Materials science, Physics

## Abstract

The increase in global energy consumption and the related ecological problems have generated a constant demand for alternative energy sources superior to traditional ones. This is why unlimited photon-energy harnessing is important. A notable focus to address this concern is on advancing and producing cost-effective low-loss solar cells. For efficient light energy capture and conversion, we fabricated a ZnPC:PC70BM-based dye-sensitized solar cell (DSSC) and estimated its performance using a solar cell capacitance simulator (SCAPS-1D). We evaluated the output parameters of the ZnPC:PC70BM-based DSSC with different photoactive layer thicknesses, series and shunt resistances, and back-metal work function. Our analyses show that moderate thickness, minimum series resistance, high shunt resistance, and high metal-work function are favorable for better device performance due to low recombination losses, electrical losses, and better transport of charge carriers. In addition, in-depth research for clarifying the impact of factors, such as thickness variation, defect density, and doping density of charge transport layers, has been conducted. The best efficiency value found was 10.30% after tweaking the parameters. It also provides a realistic strategy for efficiently utilizing DSSC cells by altering features that are highly dependent on DSSC performance and output.

Dye-sensitized solar cells (DSSCs) are a type of photovoltaic device that converts sunlight into electrical energy. Unlike traditional silicon-based solar cells, DSSCs are fabricated through a flexible manufacturing process using a sensitizing dye in order to capture light and generate electrons. These solar cells’ main benefits are their cost-effectiveness, lightweight, and feasibility for efficient light harvesting even at relatively low temperatures^1–6^. One of the key advantages of DSSCs is that they can be made from low-cost materials, making them an attractive option for large-scale solar energy generation. Abundant materials worldwide are readily available for manufacturing DSSCs, whereas traditional solar cells often rely on rare and expensive materials. This makes DSSCs a potentially feasible substitute for other types of solar cells with wide applicability: particularly, they are applicable in areas with limited resources or access to high-end technology^7–9^. Despite their potential, DSSCs still face several challenges that must be overcome before they can compete with traditional solar cells. These include improving their efficiency, stability, and durability, as well as reducing the cost of production. However, ongoing research and development in this area are making significant strides, and DSSCs continue to hold promise as a prospective alternative to traditional solar cells.

Theoretical modeling studies have evolved toward sustainable energy substitutes, energy security, and reducing pollution in constructing solar cells of practical significance. Using theoretical simulations in cell design and production can enhance experimental data and conserve time and money. SCAPS-1D is a unique, user-friendly, and intelligent software employed for modeling and analyzing third-generation solar cells^10–15^. Korir and colleagues^16^ developed a DSSC comprised of solid-state layers via numerical simulation using SCAPS-1D: they optimized numerous factors of the performance of PC61BM as the electron acceptor and reported a power conversion efficiency (PCE) of 5.38%. Jahantigh and Safikhani^17^ also employed SCAPS-1D to assess the effectiveness of DSSC under various high-temperature situations: their findings show that the CuI as hole transport layer (HTL) performs better than the other two HTLs in terms of performance and result. By using the SCAPS-1D simulation software, Noorasid et al.^18^ modeled and analyzed solid-state DSSC (ss-DSSC) paired with CuI as a hole transport material and found it compelling: after adjusting various parameters, the researchers' findings demonstrate that their designed model with back contact performs better than without back contact, owing to the lower HTL thickness. Ojotu and Babaji^19^ used SCAPS for modeling and analyzing ss-DSSC with Poly 3-Hexylthiophene (P3HT) as the HTM: after optimizing their device settings, their device got a PCE of 4.90% and a fill factor (FF) of 56.45%. Nithya and Sudheer^20^ used the SCAPS-1D program to develop an NFA-OSC. They claimed CuI was a more effective HTL than conventional arrangements. Their method obtains a PCE of 15.68% under ideal circumstances. Abdelaziz and coworkers^21^ found that the productivity of the graded bulk heterojunction (GBHJ) photovoltaic (PV) cell was higher than that of the bulk heterojunction (BHJ) PV cell: they got a PCE of 12.39% using non-fullerene acceptors. CuSCN serves as the HTL in the non-fullerene OSC that was created by Sharma and colleagues^22^ using SCAPS-1D: they adjusted the settings and got a 20.36% power conversion efficiency.

Since the very first DSSC released by O'regan and Grätzel^2^ in the early 1990s, DSSCs have sparked considerable attention. The device performance of a DSSC can be effectively improved by employing its layers optimally and consistently. The dye gets oxidized by releasing electrons, and the electrolyte compensates for the dye's oxygen loss by supplying electrons to the dye and preventing oxidation. Typically, electrolytes in the liquid state comprise a redox system (R/R) to help them conduct electricity^23^. Among these, the iodide/triiodide (I/I_3_) redox couple reigns supreme as the most prevalent choice for electrolytes in DSSCs. There are several challenges in preparing liquid electrolytes: some of them are solvent evaporation, low heat stability, and challenging sealability. To avoid this problem in the future, employing a solid-state electrolyte, such as that used in ss-DSSCs^9,24,25^, is preferable. HTLs have been used widely in DSSCs due to their remarkable features like high stability, no toxicity and no leakage of charge carriers. Also, this helps to transport holes efficiently, yielding increased productivity^26^. Typical organic and inorganic HTLs have been investigated to address the issues with liquid electrolytes, but their efficiency is still relatively low, in addition to the problems with sealing^27^. However, several organic and inorganic-based HTLs have the potential of achieving noteworthy results in light harvesting^28^. A DSSC with titania as an electron extraction layer and N719 as a photo-harvesting layer realized a PCE of 8.5%^29^. Organic hole acceptor materials such as P3HT and POT were initially used in ss-DSSSs. They displayed PCE of 1% due to inadequate polymer HTL pore filling into the mesoporous TiO_2_ film, which causes ineffective charge separation and low charge extraction efficiency^30^. The PC61BM is a well-known electron acceptor layer employed in photovoltaic cells. ITO/PEDOT:PSS/CH_3_NH_3_PbI_3_/Al cell setup with PC61BM as the electron transport layer (ETL) produces PCE of 3.33%^31^. PC61BM has a higher electron affinity as well as a suitable electron acceptor. The P3HT/PC61BM heterojunction layer transmits carriers and captures solar photons, enhancing DSSCs' photoelectric response^32^. There are lots of other studies that were reported to lift the efficacy of DSSCs^33^. Recent studies have found that the efficiency of these solar cells can approach 12% or more^34–37^.

The main aim of our study is to assess the potential of the ZnPC:PC70BM structure as a replacement for conventional dyes in DSSCs, offering favorable optoelectronic properties that could potentially improve their efficiency and durability. By analyzing the performance of this new structure, we determine its feasibility for use in commercial applications and its potential to address some of the challenges faced by conventional dyes in DSSCs. Herein, we also explored the performance of ZnPC:PC70BM-based DSSC by varying different parameters, primarily series/shunt resistances and metal contacts that enhance its effectiveness. This study offers valuable insights into the potential of the ZnPC:PC70BM structure and provides useful information for the development of more efficient and durable DSSCs. Additionally, its results were evaluated against actual and simulated data from other pieces of literature.

## Device design and simulation

### Design and method

**Figure 1:**
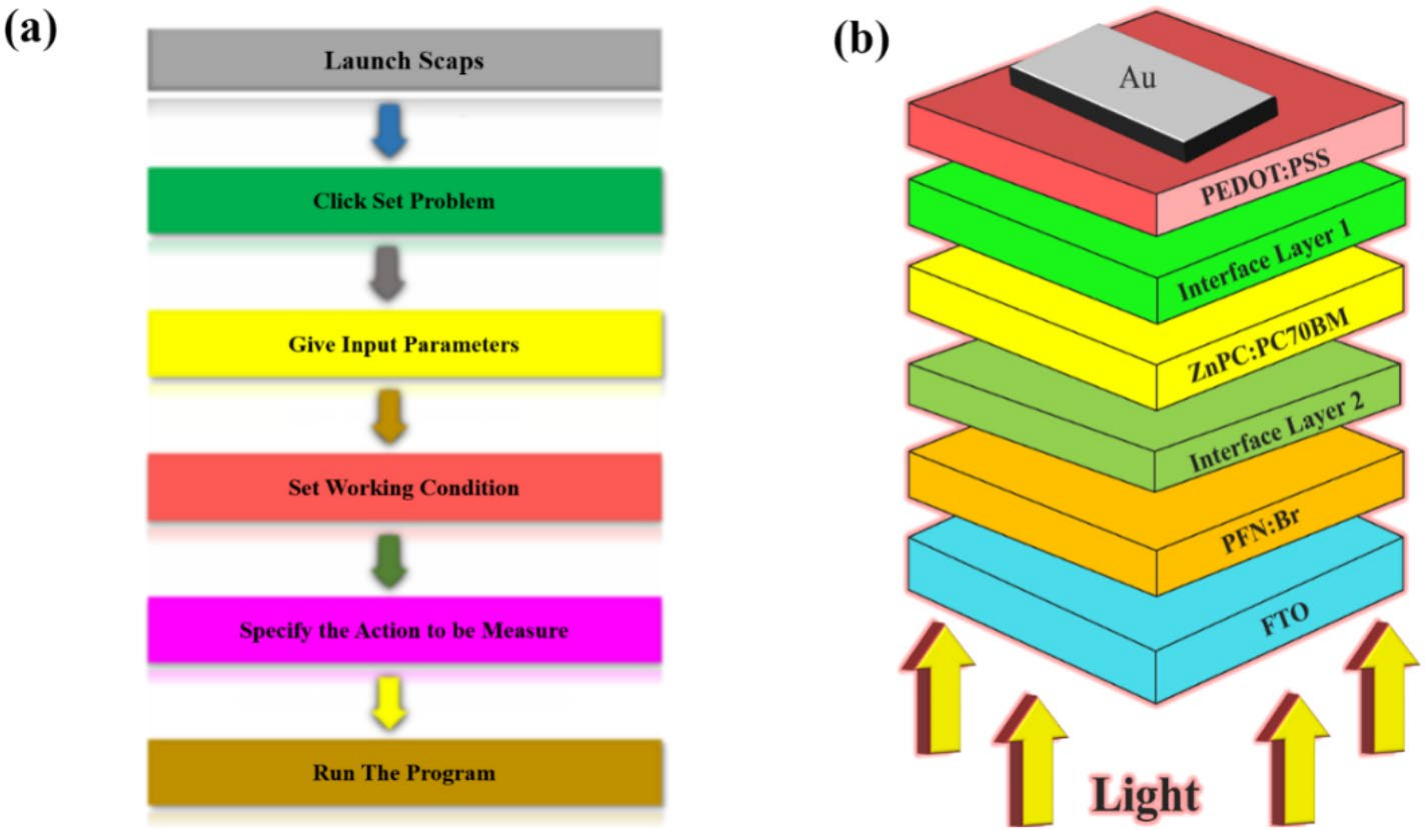
Schematic illustration: (**a**) Basic steps for numericalization SCAPS-1D, and (**b**) A diagrammatic representation of the intended DSSC structure.

The simulated cell in several segments was designed and analyzed using SCAPS (version 3.3.07)^35^. Figure 1a displays the SCAPS program's step-by-step simulation approach. SCAPS’s principal role is to solve one-dimensional semiconductor equations alongside Gummel-type iteration and Newton-Raphson differentiation methods to compute performance characteristics of designed solar cells effectively^38,39^. The necessary equations are:

1 $$\frac{\partial }{\partial x}\left( {\varepsilon_{0} \varepsilon \frac{\partial \Psi }{{\partial x}}} \right) = - q\left( {p - n + N_{D}^{ + } - N_{A}^{ - } + \frac{{\rho_{def} }}{q}} \right)$$

2 $$- \frac{{\partial J_{n} }}{\partial x} - U_{n} + G = \frac{\partial n}{{\partial t}}$$

3 $$- \frac{{\partial J_{p} }}{\partial x} - U_{p} + G = \frac{\partial p}{{\partial t}}$$

4 $$J_{n} = - \frac{{\mu_{n} n}}{q}\frac{{\partial E_{Fn} }}{\partial x}$$

5 $$J_{p} = + \frac{{\mu_{p} p}}{q}\frac{{\partial E_{Fp} }}{\partial x}$$

where $\epsilon$ is the dielectric constant, *q* is the charge on the electron, *J*_n_ and *J*_p_ are current density for electrons and holes respectively, *G* is the generation rate, $\Psi$ is the electrostatic field, *E*_*F*_ is the electric field, *U* is the rate of recombination, *x* is the thickness, *p*, and *n* are allowed concentration of holes and electrons, *N*_D_ and *N*_A_ are ionized concentration for donors and acceptors, and $\mu$ is the mobility of charge carriers.

**Figure 2:**
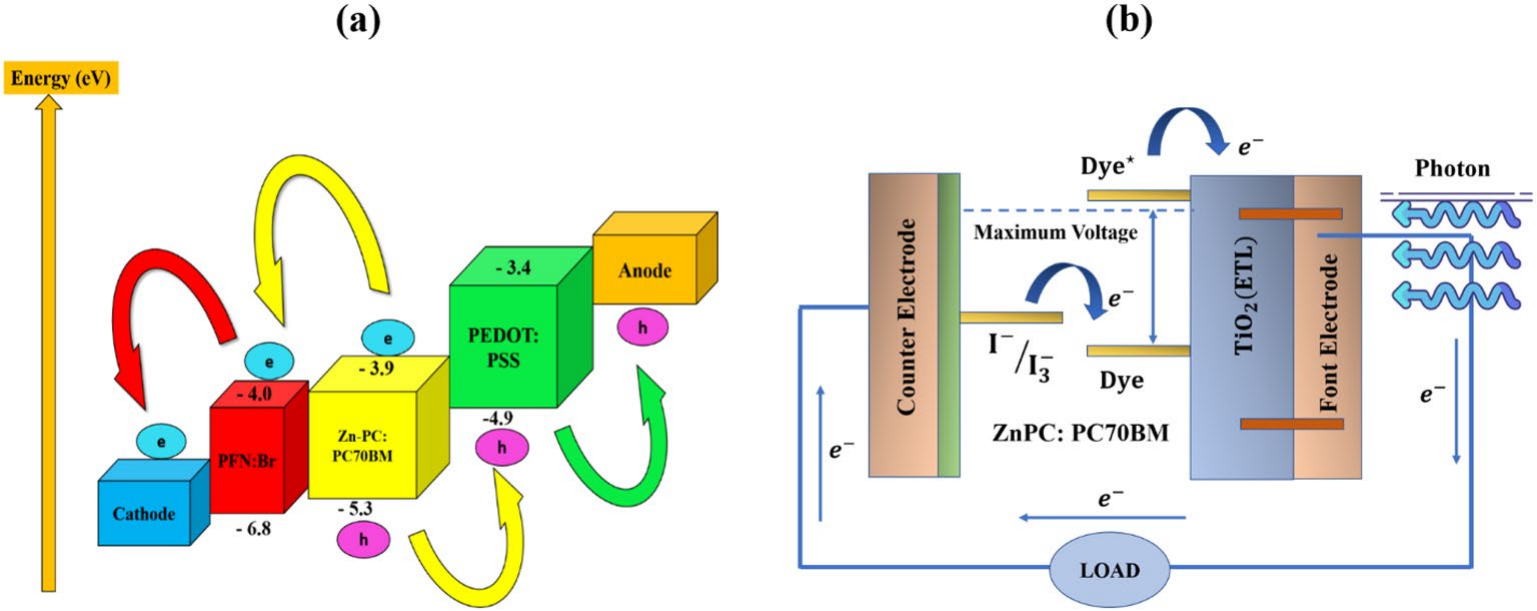
(**a**) HOMO–LUMO band diagram of the proposed DSSC, and (**b**) Schematic of the operation principle of DSSC.

This research presents a heterojunction arrangement for DSSCs using ZnPC:PC70BM (Zinc-phthalocyanine: ^6,6^-Phenyl-C71-butyric acid methyl ester) as the dye-sensitizing layer. The other layer of the device includes an HTL which is Poly(3,4-ethylene dioxythiophene)-poly(styrene sulfonate) (PEDOT:PSS), an ETL which is Poly(9,9 bis(3'-(N,N-dimethyl)-N-ethylammoinium-propyl-2,7-fluorene)-alt-2,7-(9,9-dioctylfluorene)) dibromide (PFN:Br), a transparent front electrode fluorine-doped tin oxide (FTO), and a back metal electrode gold (Au) as illustrated from Fig. 1b. The energy band diagram, so-called the highest occupied molecular orbital (HOMO)-the lowest unoccupied molecular orbital (LUMO) band diagram of the appropriate DSSC is visualized in Fig. 2a.

DSSCs have unique structural properties that set them apart from other types of photovoltaic cells. A simple photo-electrochemical cell like DSSC is a photoelectrode that combines a semiconductor material (such as TiO_2_ in many cases) and sensitized dye molecules^40,41^. We adopted ZnPC as that material in our case. This electrode, along with a catalytic counter electrode, is deposited on a transparent conducting material. The electrolyte in a DSSC is a combination of organic and inorganic materials, including inorganic salts, a redox couple, an n-type semiconductor, and conducting polymers. The dye sensitization is used to capture light and generate electron–hole pairs, while the electrolyte serves as a redox mediator to transfer electrons between the electrodes. This leads to rapid electron injection into the conduction band of the TiO_2_ semiconductor material. The transparent conducting material then transports the electrons to the counter electrode through an outer circuit. Meanwhile, the dye is oxidized at its ground state, and the electrolyte regenerates it. The operational diagram of DSSC is illustrated in Fig. 2b. Overall, the unique combination of materials and processes in a DSSC allows for the efficient conversion of light into electrical energy. The presence of sensitizing dye molecules and an electrolyte enables the cell to generate and transport charge carriers effectively, while the nanomaterials used in the photoelectrode provide a large surface area for light absorption.

However, alternative types of solar cells have been developed in recent years, which eliminate the need for dye sensitization and/or electrolyte. For example, some DSSC designs replace the liquid electrolyte with a solid-state electrolyte, which can improve stability and reduce leakage problems. In these cases, the cells may still be called DSSCs because they operate based on similar principles of light absorption and charge transfer, even if the specific components are different. The development of new and alternative materials for use as the active layer or sensitizer in DSSCs, such as metal-halide perovskites, has resulted in higher power conversion efficiencies and improved stability^42–44^. Additionally, innovative device architectures such as tandem and hybrid solar cells have shown great potential in improving DSSC performance. The ongoing progress in DSSC technology and research is expected to drive further advancements and broader adoption soon.

### Parameters for device simulation

The whole list of the simulation parameters used to model the structure’s layers were meticulously selected from the studies described in^17,20,21,45–53^. Tables 1 and 2 provide material parameters for each layer used in this simulation. Other material properties are also suitably adjusted: the thermal velocity of electrons and holes are adjusted at 10^7^ cm/s for instance. Device modeling was made more accessible by using absorption profiles for all layers in the simulation, which were acquired from several sources^48,50,54–56^. To give a more realistic picture of the device, this design comprises two interface defect layers, marked by the letters IDL1 (PFN:Br/photo-harvesting layer) and IDL2 (photo-harvesting layer/PEDOT:PSS). The AM1.5G spectrum was used to model this cell at a temperature of 300 K. The incident lamp or sun power was set at 1000 W/m^2^. Additionally, all operating point settings and numerical factors were kept at their actual value. Scanning voltage has been set to a range of 0 V to 1 V. All simulations in this software are conducted with the above values.

**Table 1:** Parameters of material configured in simulation for different layers.

Parameters	PFN:Br	ZnPC:PC70BM	PEDOT:PSS
Thickness (nm)	10	500	100
Acceptor density (cm^-3^)	0	0	${10}^{18}$^21^
Donor density (cm^-3^)	$9 \times {10}^{18}$^20^	$0$	0
Effective DOS for VB (cm^-3^)	10^19^^20^	10^19^^50^	2.5 $\times$ 10^21^^46^
Effective DOS for CB (cm^-3^)	10^19^^20^	10^19^^50^	1.7 $\times$ 10^19^^46^
Bandgap (eV)	2.8^47^	1.4^48^	1.5^21^
Relative dielectric permittivity	5^20^	3^17^	3^21^
Mobility of electron (cm^2^/Vs)	2 $\times$ 10^–6^^21^	5^17^	$1.69 \times {10}^{-4}$^49^
Mobility of hole (cm^2^/Vs)	1 $\times$ 10^–4^^21,47^	5^17^	${1.69 \times 10}^{-4}$^49^
Electron affinity (eV)	4^20^	3.9^48,53^	3.4^21^
Defect density (cm^-3^)	${10}^{9}$^47^	${10}^{12}$^22^	${10}^{9}$^21^

**Table 2:** Device parameters set in the simulation.

Interface defect density^20^	
IDL1 (ETL/Active) defect density	${10}^{9} {\mathrm{cm}}^{-2}$
IDL2 (Active/HTL) defect density	${10}^{9} {\mathrm{cm}}^{-2}$
Back metal contact properties^51,52^	
The electron work function of Au	−5.1 eV
Surface recombination velocity of the electron	${ 10}^{5}\mathrm{ cm}/\mathrm{s}$
Surface recombination velocity of hole	${10}^{7}\mathrm{ cm}/\mathrm{s}$
Front metal contact properties^51,52^	
The electron work function of TCO	−4.4 eV
Surface recombination velocity of the electron	${10}^{7}\mathrm{ cm}/\mathrm{s}$
Surface recombination velocity of hole	${ 10}^{5}\mathrm{ cm}/\mathrm{s}$

## Simulation findings and discussions

### Photoactive current density and quantum efficiency response

In this study, we employed ZnPC:PC70BM as an absorber material for DSSC, wherein ZnPC acts as a donor component and PC70BM as an acceptor component. The hybrid material ZnPC:PC70BM results in efficient dissociation of electron–hole pair. ZnPC:PC70BM exhibits low electronic bandgap and lower reorganization energy, which results in more charge transfer and higher optical conductivity. As a result, their optoelectronic characteristics are more delicate. According to the findings, ZnPC:PC70BM can be a workable replacement as a conventional dye for DSSCs and might even be used to create DSSCs for future generations.

**Figure 3:**
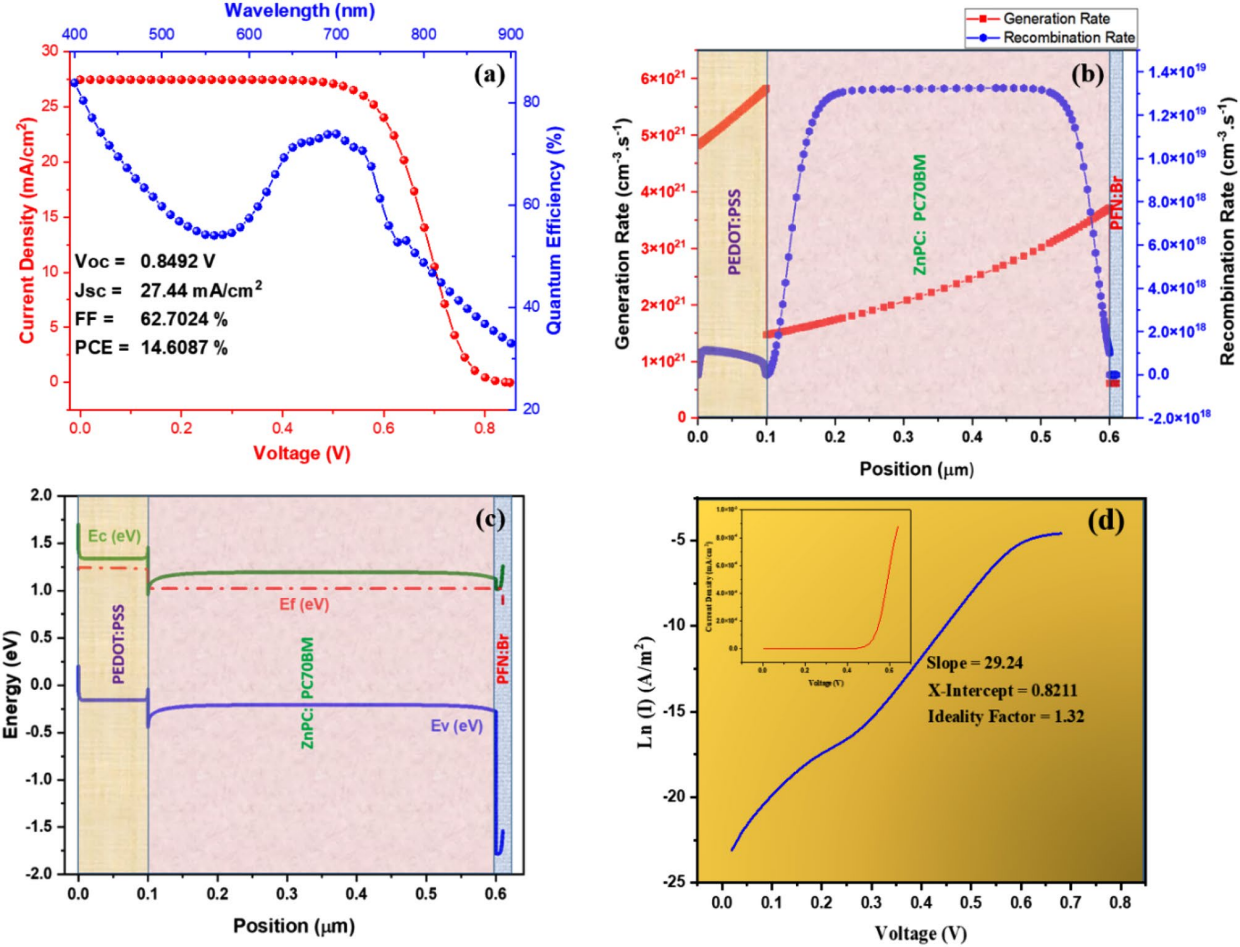
(**a**) J–V and QE curve of the intended device (**b**) Generation and recombination rate of photogenerated carriers in device (**c**) Energy band structure of purposed device, and (**d**) Ln (I) vs voltage with an inset representing dark J–V measurement.

The numerical analysis is performed on ZnPC:PC70BM-based DSSC, with an active layer thickness of 500 nm. The materials parameters for the absorber and other supporting layers are mentioned in Table 1. The series resistance (Rs) and shunt resistance (Rsh) values are set at 1 Ωcm^2^ and 1000 Ωcm^2^. A low Rs and a high Rsh are desirable in a PV cell because they help to minimize power losses due to resistance and prevent localized hot spots and current leakage paths that can reduce cell efficiency and reliability. The current density–voltage (*J–V*) and the quantum efficiency (*QE*) curves are illustrated in Fig. 3. The open-circuit voltage (*V*_oc_) refers to the measure of recombination and is calculated when zero current flows in a device, and its value was 0.85 V at 500 nm. While short-circuit current density (*J*_*sc*_) is the maximum current drawn from the photovoltaic cell and it is measured when the voltage is zero. The *J*_*sc*_ and other output parameters like PCE and FF obtained at 500 nm were 27.44 mA/cm^2^, 14.61% and 62.70%. Resistive losses in photoactive and charge-transporting layers are responsible for the modest inflection at the end of the *J–V* curve. This indicates the presence of a series resistance (Rs), which is shown by a negative value for the inverse slope of the current–voltage curve near voltage end. The QE graph of a solar cell is expected to have a rectangular or square shape, but this ideal shape can be reduced by various factors such as recombination, reflection losses, and surface passivation. The observed variability in the QE spectra, as seen in Fig. 3a, can be attributed to these losses. The QE can be determined by the charge carriers being transported and collected by the electrodes. In this particular case, front surface recombination, low diffusion length, and reflection losses result in a drop in QE from 400 to 550 nm. However, the QE rises again from 550 to 700 nm due to a significant increase in charge carrier production from light absorption, which improves the collection probability and QE of the device. The analysis shows that the mean QE obtained for a 500 nm thickness of the absorber layer is 70%.

It is also important to note that the QE is a measure of the efficiency of the solar cell at converting photons into charge carriers (electrons and holes), while the Jsc is a measure of the maximum achievable current density. The two values are related but not identical, and it is possible for a cell to have a high Jsc but a lower QE if there are losses in charge collection or other factors that reduce the efficiency of the conversion process.

Figure 3c depicts the band-alignment of the proposed device structure. Excitons are produced when sunlight strikes the photoactive material (ZnPC:PC70BM). Excitons are confined electron–hole pairs that must be separated to transport and extract charge carriers effectively. Two different materials with suitably matched band levels are utilized to isolate the excitons. These materials are referred to as donor and acceptor. At the heterojunction of these materials, excitons are commonly separated, allowing free electrons and holes to be transmitted by charge-carrying layers (i.e. PFN:Br and PEDOT:PSS). To maximize charge carrier extraction at the electrode, the HOMO level of HTMs should be greater than the valence band of the absorber layer for hole extraction.

Figure 3b depicts the device's carrier production and recombination rates due to optical excitation. Since the light entered from ETL side, the generation and recombination rates of carriers in the cell are lowered from the ETL side to the HTL side in the photo-harvesting layer. The recombination rate is relatively high in the photoactive material due to many defects. Despite this, photo-generated carriers are abundant due to the absorber material's narrow bandgap and considerable thickness. That results in increased absorption and an improvement in the performance characteristics of a cell.

DSSCs are a promising cell technology for converting sunlight into electricity. One important parameter that affects the efficiency of DSSCs is the ideality factor, which is a measure of how closely the cell's behavior matches ideal photovoltaic behavior. In a DSSC, the ideality factor is related to the recombination of charge carriers in the cell. When a photon is absorbed by the dye in the cell, an electron is excited and moves into the conduction band of the semiconductor. The electron then travels through the semiconductor to the electrode, where it can be collected as current. However, some of the electrons may recombine with holes before they reach the electrode, which causes a reduction of the cell efficiency. Figure 3d represents dark J–V measurements for the determination of ideality factor.

The ideality factor can be determined experimentally by measuring the current–voltage characteristics of the DSSC and fitting the data to the equation^57,58^:

6 $$I = I_{0} {\text{exp}}\left( {\frac{qV}{{NkT}} - 1} \right)$$

where I is the current, I_0_ is the saturation current, q is the elementary charge, V is the voltage, N is the ideality factor, k is the Boltzmann constant, and T is the temperature. The ideality factor can be obtained from the slope of the plot of Ln(I) vs V, and it quantifies how many charge carriers recombine during this procedure. In other words, the ideality factor quantifies the level of charge carrier recombination and, consequently, related to determining how efficiently a DSSC transforms sunlight into energy. In our intended design, the ideality factor was 1.32.

### Influence of photoharvesting layer thickness on cell functionality

**Figure 4:**
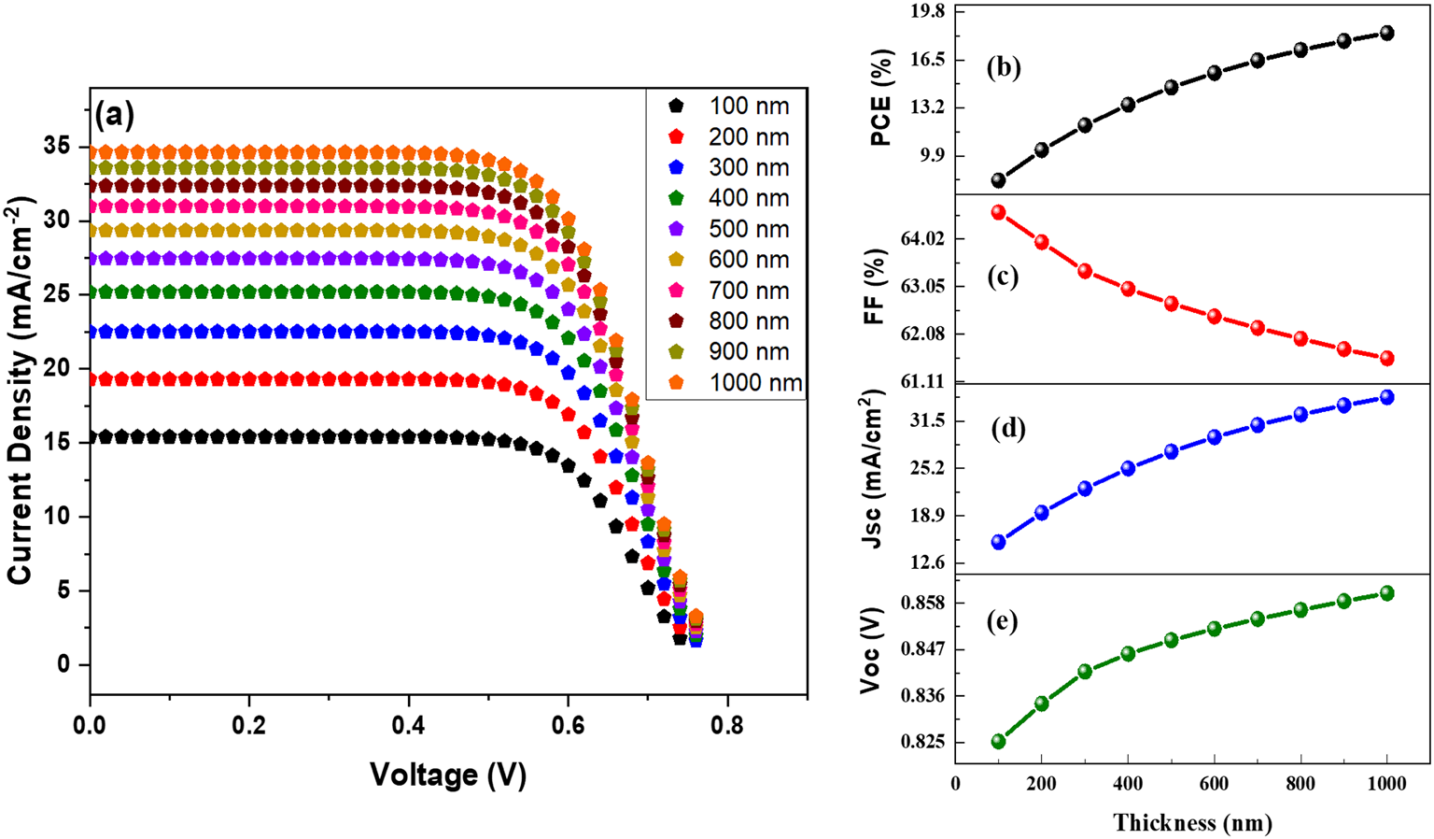
(**a**) J–V comparison at different values of thickness, (**b**) PCE, (**c**) FF, (**d**) Jsc, and (**e**) Voc in relation to photoactive layer thickness.

The photoactive layer of any PV cell is critical to the device's operation and output. This study increased the thickness of the harvesting layer from 100 to 1000 nm. Keeping all other variables constant allowed us to investigate how this variance affected the device results. The J–V characteristics and the effect of increasing thickness on the DSSC performance parameters are shown in Fig. 4. Figures 4a–e show that there is a relationship between the variety in device outcomes and the depth of the active material. The J–V curve is remarkably enhanced initially as the thickness of the photoactive material increases. This is attributed to the abundant formation of charge carriers. However, as the thickness continues to increase, the degree of improvement in the J–V curve begins to diminish. This is due to the increment in the recombination rate; such an increase is potentially surpassing the generation rate, leading to a decline in the carrier diffusion length.

The Jsc, Voc, and PCE values increase noticeably when the absorber layer thickness increases from 100 to 500 nm. The increase in the concentration of electron–hole pairs in the photoactive layer causes this. As the thickness of the photoactive layer increases from 500 to 1000 nm, it will take longer for the produced carrier to reach the appropriate electrodes, resulting in a higher low diffusion and low generation rate. Which causes the *J*_*sc*_ and *V*_*oc*_ to increase steadily. The largest *J*_*sc*_ and *V*_*oc*_ achieved is 34.64 mA/cm^2^ and 0.86 V when the device's thickness is 1000 nm. The fill factor reduces from 64.56% to 61.58% as the thinness of an absorber material grows to 1000 nm. When the FF is high, the cell can supply all of the energy it produces to the electrical demand. That might be because the absorber material is relatively thick, which boosts the cell's series resistance and increases the rate at which the cell's internal power is depleted. Therefore, the appropriate thickness value is necessary to yield better outcomes, and the low thickness makes a cost-effective PV cell.

### Influence of series and shunt resistance on cell functionality

Resistive losses, which often takes place by carrier recombination and current leakage, limit the performance of DSSCs. Shunt resistance and series resistance are responsible for these losses. The behavior of the *J–V* curve has impacted due to these parasitic losses. The term "internal resistance" (*R*_*i*_) referred to the resistance present in PV cells and caused by the materials, electrodes, and interface barriers^59,60^. *R*_*i*_ was predominantly driven by the electrical resistance associated with the contacts, and the electrical loss was present in both the active and supporting layers. The series resistance (*Rs*) has little impact on *V*_*oc*_ but significantly impacts FF and PCE. Furthermore, it lowers *J*_*sc*_. Therefore, during real-cell production, it is essential to conduct structural optimization by studying the impact of series and shunt resistances on cell performance. Previous studies have shown that increasing *R*_*s*_ causes a decrease in *J*_*sc*_ but has no effect on *V*_*oc*_ in a solar cell. The *R*_*sh*_ is determined by device architecture, which includes charge recombination mechanisms (edge effects) such as active layer pinholes and recombination losses^60,61^.

Consequently, low *R*_*sh*_ causes a loss in photovoltage and may also have a slight effect on the collected photocurrent. In this investigation, we have varied the series and shunt resistance from 1 to 5 Ωcm^2^ and 100–600 Ωcm^2^ to observe the influence on device performance and output parameters. Under ideal illumination, the typical *J–V* characteristic of a photovoltaic cell may be characterized using the Shockley equation.

7 $$J_{SC} = J_{0} \left[ {exp\left( {\frac{{e\left( {V - JR_{S} } \right)}}{NkT}} \right) - 1} \right] + \frac{{V - JR_{S} }}{{R_{SH} }} - J_{Ph}$$

where *J*_*Ph*_ denotes photocurrent density, *J*_0_ denotes dark saturation current, *T* is room temperature, and *e* denotes charge on an electron. In ideal conditions, *R*_*s*_ would be 0, and *R*_*sh*_ would be limitless.

**Figure 5:**
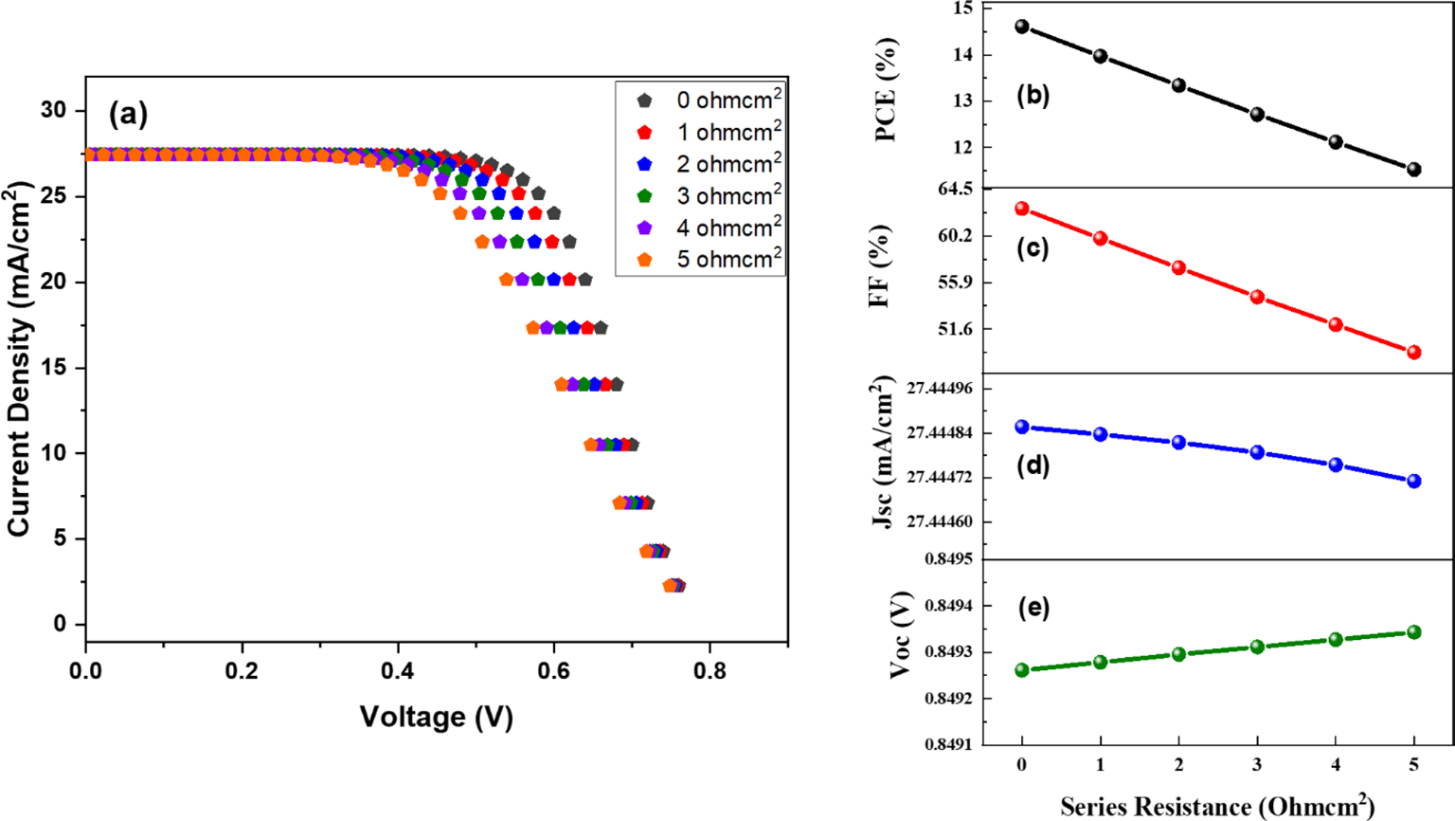
(**a**) J–V comparison at different values of series resistance, (**b**) PCE, (**c**) FF, (**d**) Jsc, and (**e**) Voc in relation to series resistance.

**Figure 6:**
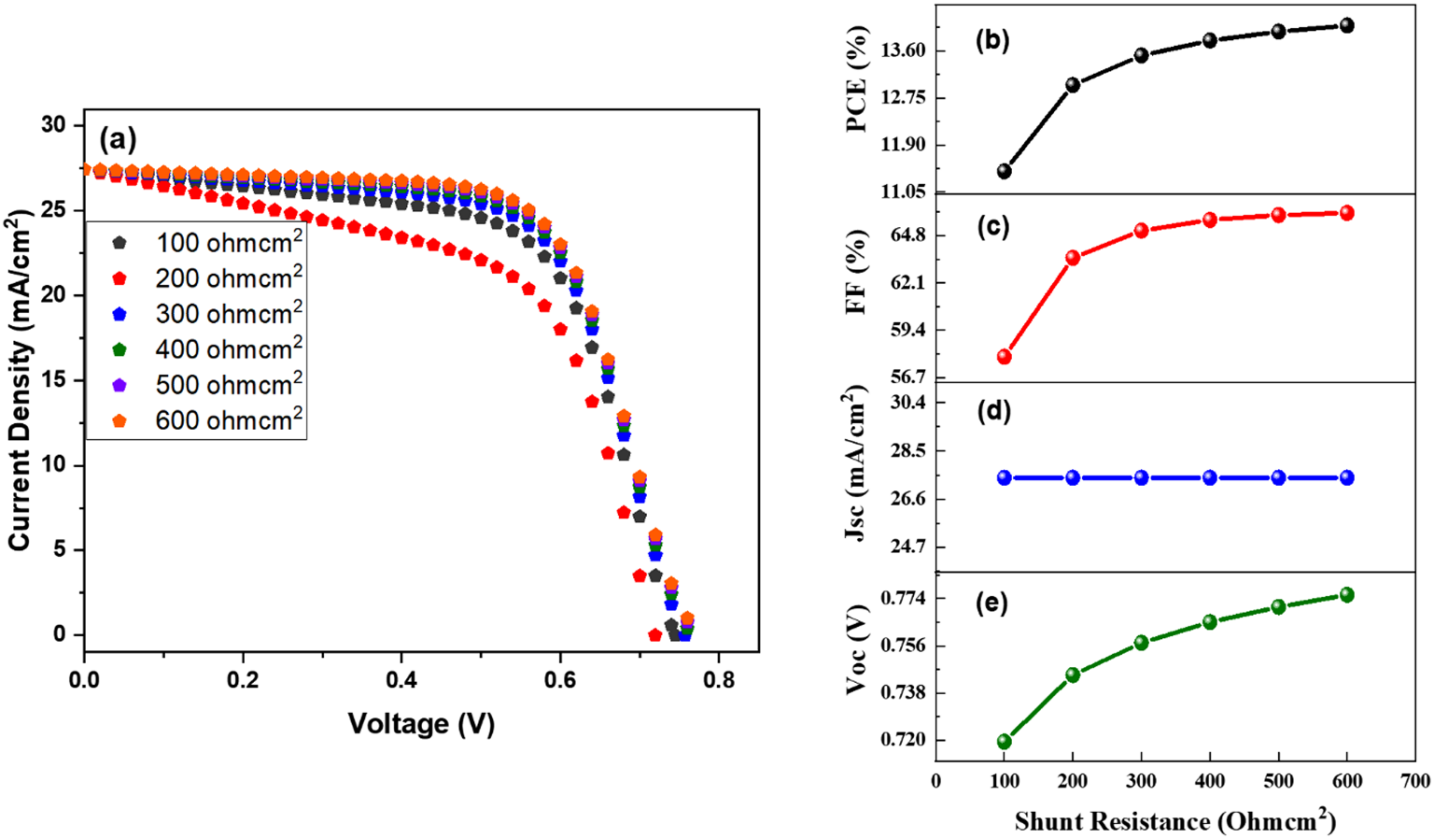
(**a**) J–V comparison at different values of shunt resistance, (**b**) PCE, (**c**) FF, (**d**) Jsc, and (**e**) Voc in relation to shunt thickness.

Figures 5a–e and 6a–e demonstrate the J–V aspects and the influence of Rs and Rsh on the DSSC performance parameters, respectively. The J–V curve shows that an increase in series resistance causes a drop in the J–V curve since it is measured from the slope of the J–V curve at the voltage end while Rsh’s J–V curve has improved due to its quick rise. The slope of the J–V curve towards the current end is used to compute Rsh. Figure 5b–e show that when series resistance increases, PCE and FF decline considerably from 14.61 to 11.52% and 62.70–49.46%, respectively. Simultaneously, a slight change in Voc and Jsc values has been detected. The decline in the value of PCE may be ascribed to the impediment provided by series resistance to charge carrier transfer.

As a result, the carrier diffusion length shortens. These power losses add to parasitic (electrical) resistance losses. Consequently, the value of series resistance should be as low as feasible in order to obtain effective output from the cell. Figure 6b–e show that when shunt resistance increases, PCE, FF, and Voc increase dramatically from 11.42 to 14.06%, 57.88–66.09%, and 0.72–0.77 V, respectively. In contrast, a negligible influence on Jsc value has been seen. The improvement in DSSC performance parameters is due to an increase in shunt resistance value, which increases the generation rate via the reduction of recombination losses. It has come to the conclusion that the right and optimum value of Rs and Rsh is critical for increasing production.

### Influence of active layer defect density on cell functionality

**Figure 7:**
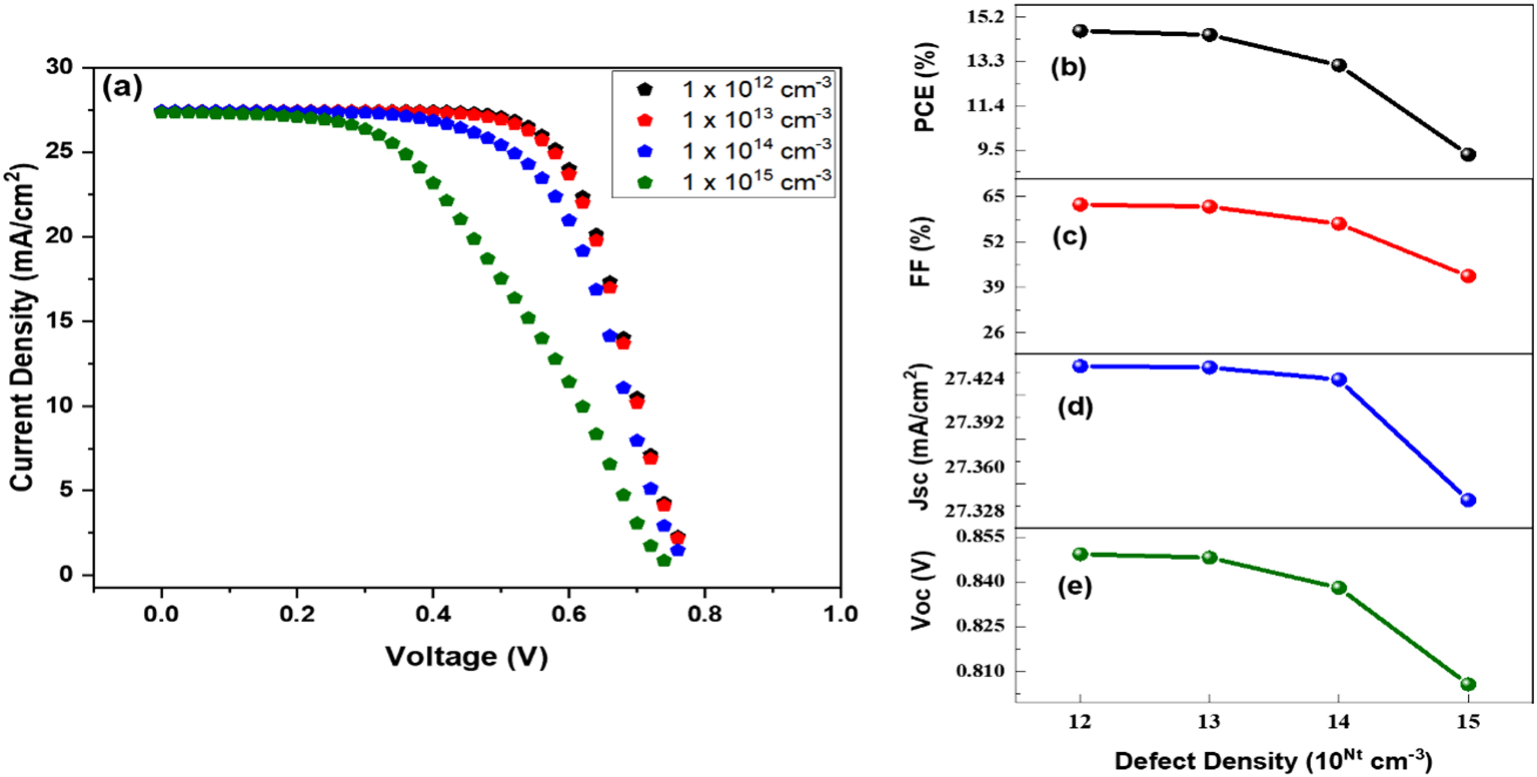
(**a**) J–V comparison at different values of defects, (**b**) PCE, (**c**) FF, (**d**) Jsc, and (**e**) Voc in relation to defects.

The quality and structure of the light-harvesting layer significantly influence the device's performance for any generation of solar cells. That is because the light-harvesting layer absorbs light in the form of photons. The poor film quality and characteristics increase the number of defect states and the rate of recombination, which reduces device performance. It is essential to the endeavor’s success that the device has a low defect density (see Fig. 7).

In active materials, trap states increase as the defect concentration grows. Trap states are localized energy levels within the bandgap of the active layer material, where electrons or holes can get trapped. When an electron or hole gets trapped in a trap state, it cannot contribute to the generation of electricity. This leads to a reduction in the carrier lifetime and an increase in recombination, which reduces the efficiency of the solar cell. The effect of trap states on the performance of a solar cell depends on their concentration and energy level. High concentrations of trap states, as well as trap states with energy levels close to the band edges, can have a significant impact on the performance of the solar cell.

Figure 7b–e illustrate the output device's performance in relation to defect density (*N*_*t*_), and Fig. 7a represents the *J–V* curve at diverse values of defect concentration of a photoactive layer. With increasing *N*_*t*_, carrier lifetime reduces, resulting in a higher recombination rate, negatively influencing output performance. It has demonstrated that a low defect density value leads to improved cell productivity. In that case, fewer traps are present in the absorber layer, and the generation rate is relatively high. In contrast, excessive defect concentrations develop additional recombination centers and traps, degrading the device's overall performance. It is feasible to enhance the device's output by lowering the defect density below 10^15^ cm^-3^, which results in a higher current density. By altering the defect density from 10^12^ to 10^15^ cm^−3^, the device output parameters PCE, FF, *J*_*sc*_, and *V*_*oc*_ reduced from 14.60 to 9.29%, 62.70–40.20%, 27.44–27.35 mA/cm^2^, and 0.85–0.80 V.

### Impact of carrier mobility of photoactive layer on cell functionality

One of the necessary factors in enhancing the productivity and outcomes of DSSCs is charge carrier mobility. The average mobility of charge carriers describes how easily charge carriers are moving from one place to another without trapping, described by a relation.

8 $$\mu_{e} \left( n \right) = \frac{{\mu_{e}^{o} n_{free} }}{{n_{free} + n_{trap} }}$$

**Figure 8:**
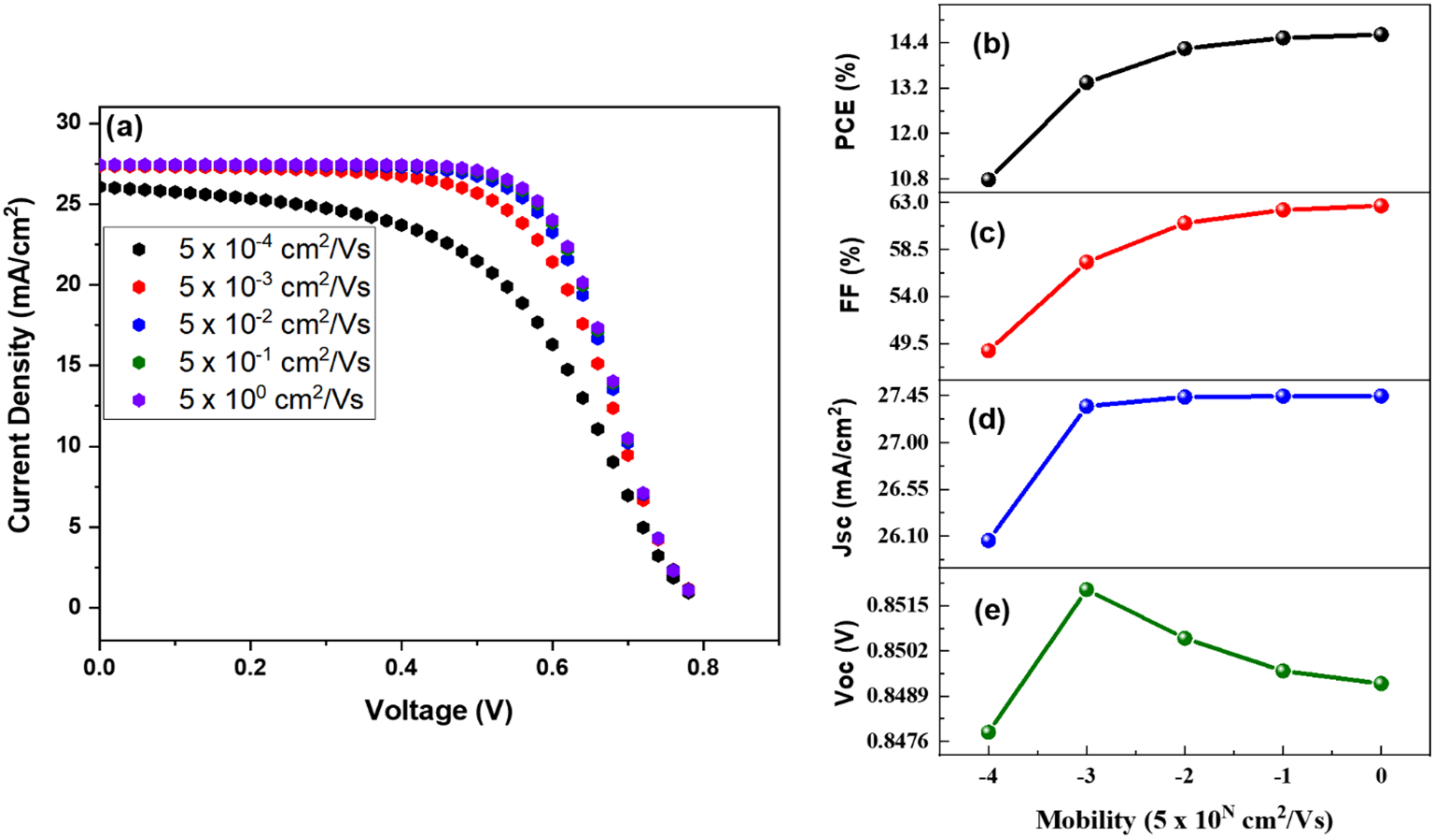
(**a**) J–V comparison at different values of carrier mobility, (**b**) PCE, (**c**) FF, (**d**) Jsc, and (**e**) Voc in relation to photoactive layer mobility.

In this work, charge carrier mobility was altered from 5 × 10^–4^ to 5 cm^2^ /Vs to investigate the impact on device performance. It has been determined that the optimum power conversion occurs at carrier mobility of 5 cm^2^ /Vs. The outcomes and *J–V* characteristics of DSSC at various charge carrier mobility values has shown in Fig. 8a–e. Short-circuit current density decreases as carrier mobility reduces due to dissociation probability, reducing efficiency and FF. When carrier mobility improves, *J*_*sc*_ increases, potentially leading to high efficiency and high FF. While Voc rises as mobility advances to 5 × 10^–3^ cm^2^/Vs, it subsequently falls as charge carrier transport improves due to less internal power depletion and a destabilized electric field in the depletion area.

### Influence of HTL and ETL layer thickness on cell functionality

The charge transport layers serve as a crucial component in facilitating the extraction and transport of holes and electrons from the active layer to the electrode. For this reason, the HTL and ETL directly affect the performance of a solar cell. The performance is mainly determined by the thickness of the HTL and ETL. The HTL and ETL are responsible for collecting and transporting the holes and electrons generated by the absorption of light.

**Figure 9:**
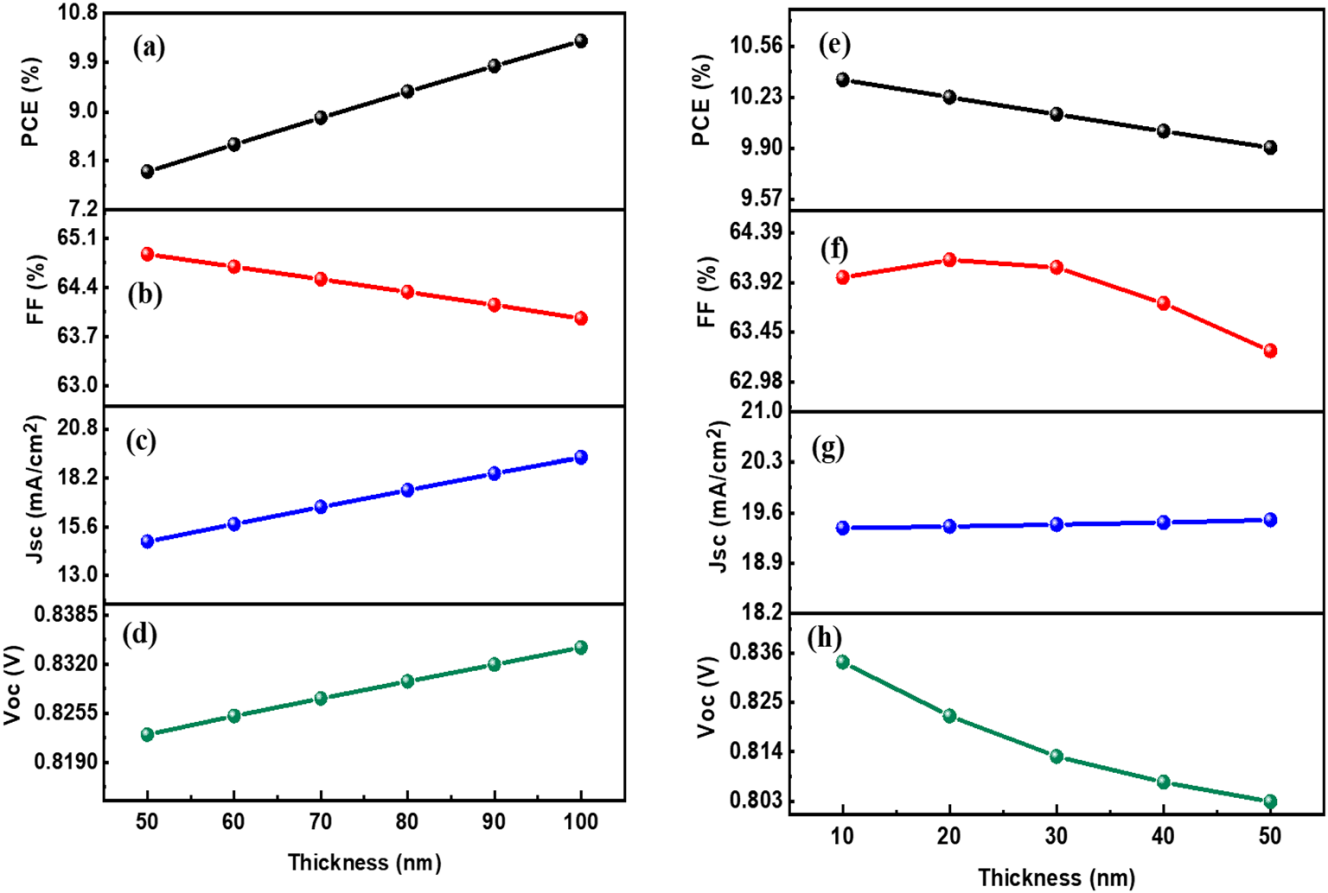
Parameters in relation to thickness of HTL layer (left panels): (**a**) PCE, (**b**) FF, (**c**) Jsc, and (**d**) Voc. Parameters in relation to thickness of ETL layer (right panels): (**e**) PCE, (**f**) FF, (**g**) Jsc, and (**h**) Voc.

In determining the performance of DSSCs, the optimal selection of the charge transport layers is important. By carefully choosing the hole transport material (HTM) and electron transport material (ETM), one can enhance the efficiency of charge transmission and collection at the electrodes. In this particular investigation, PEDOT:PSS and PFN:Br were employed as the HTL and ETL due to their promising optical and electrical characteristics. To comprehend the impact of HTM thickness on output parameters, the thickness of PEDOT:PSS was varied from 50 to 100 nm. Notably, as the thickness of the HTM layer increased, there was a substantial improvement in the central output parameters (i.e., PCE, Voc, and Jsc). This enhancement can be attributed to the exceptional charge transport properties exhibited by the HTL, which facilitates more efficient charge transfer. Additionally, the increased thickness resulted in better interaction between the HTL and the absorber layer, further augmenting the performance of the DSSCs. The grave impact on the output parameters can be clearly recognized in Fig. 9a–d. However, it is worth noting that the increase in HTM thickness had an adverse effect on FF. As you can see, FF declines as the thickness of the HTM layer increases. This outcome suggests that while the charge transport properties were improved, there might have been an increase in charge recombination or resistance within the device, leading to a reduction in FF.

ETL thickness directly affects the distance that electrons must travel to reach the electrode. As the ETL thickness increases, the path length for electron transport also increases. This can result in increased carrier recombination losses, as carriers have a higher probability of recombination before reaching the electrode. The thickness of the ETL contributes to the overall series resistance of the solar cell. It hinders the charge carriers' flow through the ETL. This can result in higher resistive losses, reducing the effective current output of the solar cell. Therefore, an optimal ETL thickness is required in order to minimize the series resistance and maximize the power conversion efficiency. Therefore, an excessively thick ETL can hinder efficient charge extraction, leading to reduced photocurrent and overall device performance, as observed in Fig. 9e–h. The ETL thickness can affect the optical absorption characteristics of the solar cell. Thicker ETLs may absorb more incident light, thereby reducing the amount of light reaching the active layer for photoconversion. This can decrease the overall photocurrent generation, leading to lower device performance. In practice, the optimal HTL and ETL thickness depends on various factors, such as the specific materials used, device architecture, and fabrication techniques. It often requires empirical optimization and device engineering to determine the ideal thickness for a given solar cell configuration.

### Influence of HTL and ETL layer defect density on cell functionality

The defect density of the HTL and ETL in a solar cell has a notable impact on its performance. Defects in the HTL and ETL can arise from various sources, such as impurities, structural imperfections, or fabrication processes, and can significantly affect the charge transport and recombination processes within the device.

**Figure 10:**
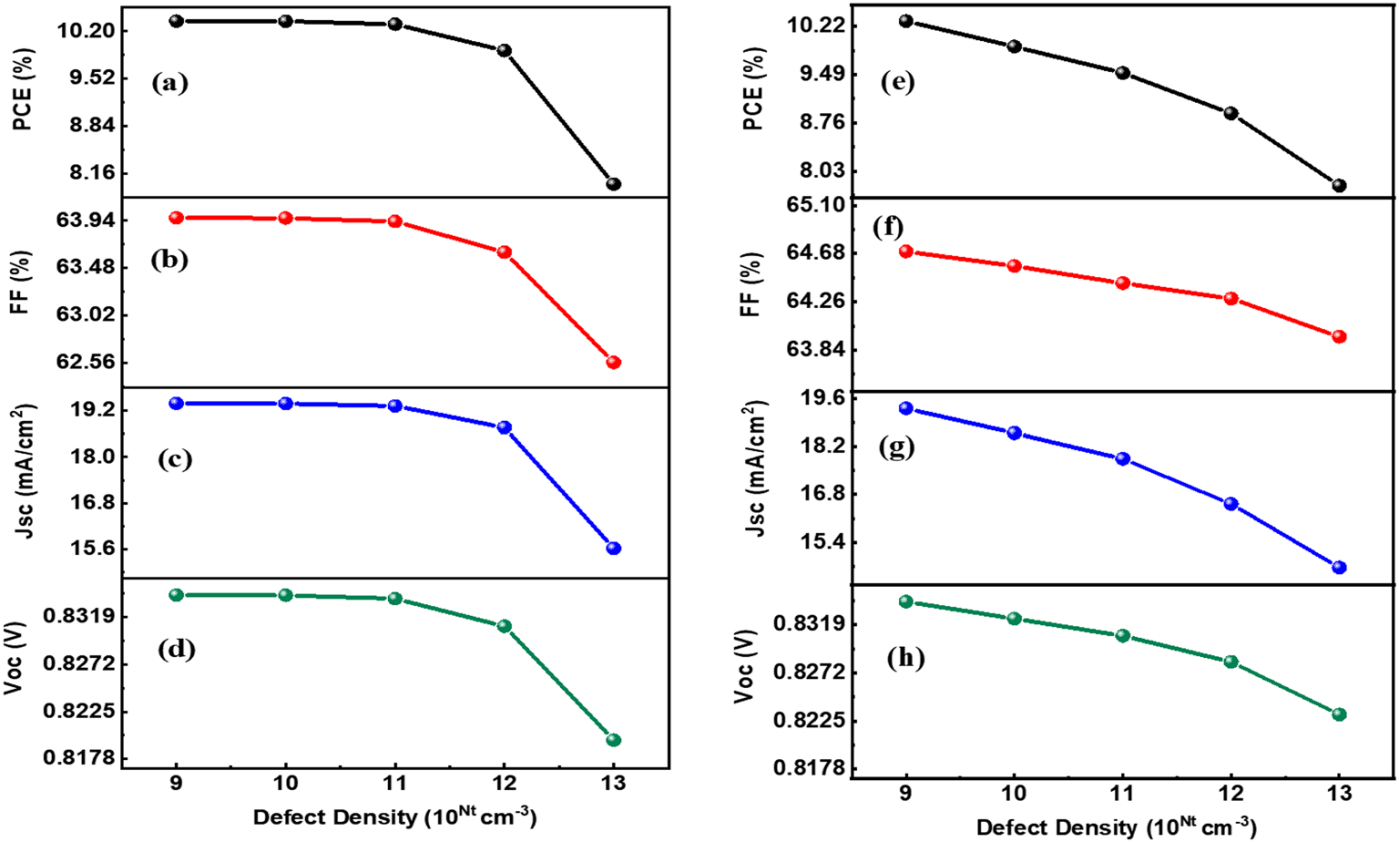
Parameters in relation to defect density of HTL layer (left panels): (**a**) PCE, (**b**) FF, (**c**) Jsc, and (**d**) Voc. Parameters in relation to defect density of ETL layer (right panels): (**e**) PCE, (**f**) FF, (**g**) Jsc, and (**h**) Voc.

Figure 10 illustrates the variation in the characteristics of DSSCs when the defect density of HTL (see Fig. 10a–d) and ETL (see Fig. 10e–h) is altered. The visual representation reveals that a lower trap density of HTL and ETL yields more favorable outcomes for cell performance. This is primarily due to the presence of fewer traps and a higher growth rate under such conditions. Consequently, both the Voc and Jsc experience an increase, leading to higher PCE and FF. Also, higher defect densities result in the generation of more capturing states, which exacerbates recombination processes within the device. As a result, the overall performance of the DSSCs is compromised. To achieve optimal results, it is recommended to maintain a defect density of 1 × 10^9^ cm^−3^ for both the HTL and the ETL. This particular defect density value strikes a balance between minimizing trap-related losses and maximizing charge transport efficiency, thereby optimizing the performance of the DSSCs.

The defect density of the charge transport layers significantly influences solar cell performance by affecting charge transport, recombination pathways, series resistance, and carrier selectivity. Minimizing defects and optimizing the quality of the HTL and ETL﻿ is crucial for achieving high-efficiency solar cells with improved charge extraction, reduced recombination losses, and enhanced overall device performance. To optimize DSSC performance, it is essential to minimize the defect density in the charge transport layers. Techniques such as careful material selection, improved fabrication processes, and defect passivation strategies can help reduce defect densities. Additionally, advanced characterization techniques can be employed to identify and quantify the nature and impact of defects in the HTL and ETL﻿, aiding in the development of strategies to mitigate their adverse effects.

### Influence of HTL and ETL layer doping density on cell functionality

The doping density of the HTL and ETL in a solar cell can have a significant influence on its performance. The HTL is typically a p-type semiconductor material responsible for transporting holes from the active layer to the electrode, while the ETL is an n-type semiconductor material that facilitates the movement of electrons from the light-absorbing layer (e.g., the active layer or absorber) to the electrode. The doping density of the HTL and ETL affects their electrical conductivity and energy level alignment with the adjacent layers.

**Figure 11:**
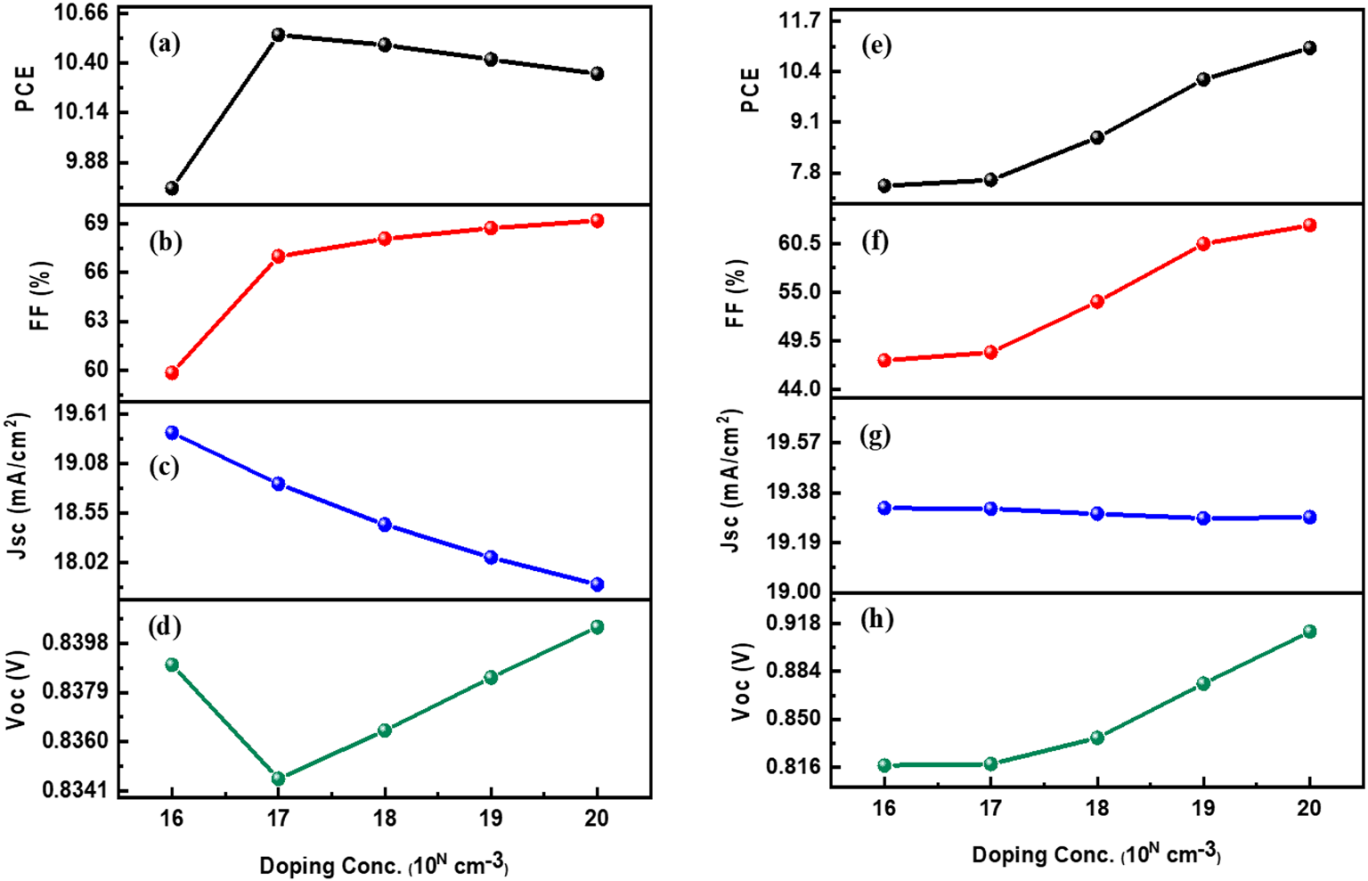
Parameters in relation to doping density of HTL layer (left panels): (**a**) PCE, (**b**) FF, (**c**) Jsc, and (**d**) Voc. Parameters in relation to doping density of ETL layer (right panels): (**e**) PCE, (**f**) FF, (**g**) Jsc, and (**h**) Voc.

In this study, the influence of HTM and ETM layer doping density on solar cell performance was investigated. We varied the doping concentration of the HTM layer from 1 × 10^16^ to 1 × 10^20^ cm^−3^ and observed its effect on the device’s performance, as depicted in Fig. 11a–d. The results indicated that increasing the doping concentration of the HTM layer had a positive impact on the solar cell's output performance. Specifically, it was found that, as the doping concentration increases, the FF and PCE of the solar cell improve. However, there was an initial decrease in Jsc and Voc. This phenomenon can be attributed to a decrease in carrier lifetime and an increase in the recombination rate at the interface between the HTM layer and the absorber layer.

In addition, the ETM layer doping enhances the device's performance when its density is altered from 1 × 10^16^ to 1 × 10^20^ cm^−3^. Doping the ETL helps to enhance charge carrier mobility, i.e., the ability of charge carriers to move through the material. Higher doping densities generally result in improved electron mobility, enabling faster and more efficient charge transport across the ETL. This, in turn, reduces carrier recombination losses and increases the overall current collection efficiency and performance of the DSSC, as illustrated in Fig. 11e–h. A slight decline in Jsc value could also be observed after increasing doping concentration. When the doping density of the ETL is raised, the density of available charge carriers (electrons) also increases. This higher concentration of carriers can enhance the likelihood of carrier recombination processes occurring within the ETL itself. The increased doping density can exacerbate carrier recombination rates, thus leading to a decline in Jsc. Furthermore, a higher doping density in the ETL can create a greater potential barrier for electron extraction at the interface between the ETL and the absorber layer. This barrier arises from the energy level alignment between the ETL and the absorber layer, which can be influenced by the doping density. If the potential barrier becomes too high, it can impede the efficient extraction of electrons from the absorber layer into the ETL, resulting in reduced Jsc.

Hence, a well-adjusted balance between the doping density and other device parameters is necessary to optimize the solar cell's performance in terms of performance parameters. After careful analysis, we determined that an optimized doping density value for the HTM and ETM layers could be 1 × 10^18^ cm^−3^ and 9 × 10^18^ cm^−3^, respectively. This particular doping concentration demonstrated several benefits. Firstly, it increased the cell conductivity, leading to a reduction in internal power depletion and series resistance. The decrease in series resistance subsequently contributed to improved PCE, FF, and Voc. These findings shed light on the significance of carefully selecting the doping density of the HTM and ETM layers to optimize the overall efficiency of solar cells.

### Influence of varying back metal contact on device performance

The work function is the minimum amount of energy required to move an electron from a metal surface to vacuum. The purpose of the contacts is to start conduction in an external circuit. According to reports^62–64^, an increase in the work function's value can boost the efficiency of PV cells. This is explained by the fact that the majority carrier's barrier height decreases as the work function value increases, resulting in ohmic contact. As a result, an increment of the metal's work function value raises the open circuit voltage and energy conversion efficiency. In this research, simulations had conducted to narrow the search for an appropriate earth-abundant metal that may serve as the back contact in the suggested device configuration. Previous research has found that DSSCs rely on metallic connections for dye regeneration, which indicates that the device performance is likely to be favorable for silver (Ag) and gold (Au). These are two most used metals with work functions of 4.7 eV and 5.1 eV and have been considered as suitable materials for dye regeneration to overcome the restrictions associated with the use of liquid electrolytes and to avoid the use of expensive HTLs.

However, due to the high cost of these materials, their use in producing solar cells that are sold at affordable prices is limited. Despite this, we have investigated the viability of many alternative metallic materials as feasible and affordable solar cell device components during this research. Simulations have been run on copper, silver, iron, platinum-oxide, carbon-copper, and gold potential dye-regenerating metallic contacts.

**Figure 12:**
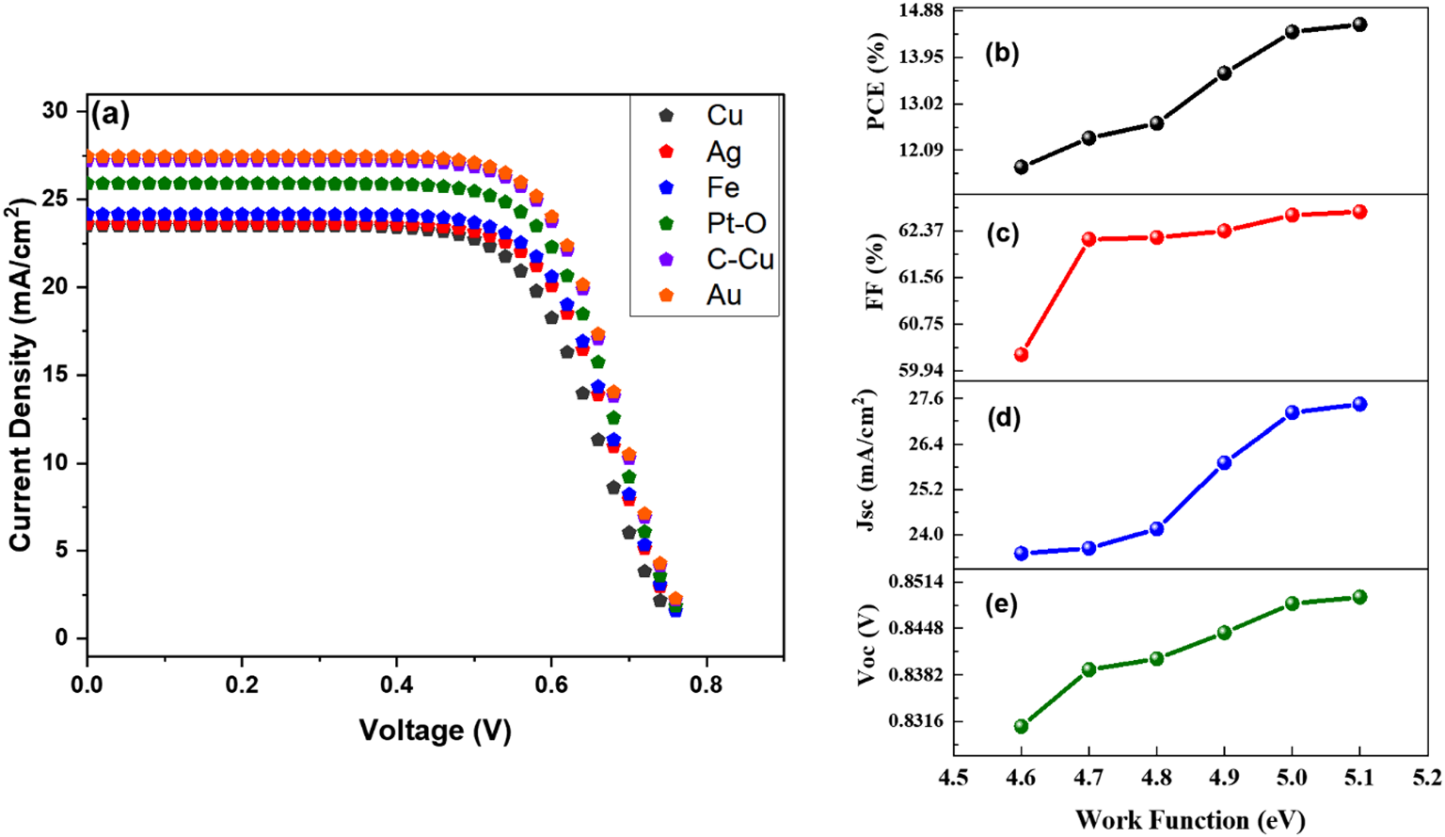
Parameters in relation to metal work function. (**a**) J–V comparison at different values of work function, (**b**) PCE, (**c**) FF, (**d**) Jsc, and (**e**) Voc.

Figure 12a–e depict the effect of J–V characteristics and the influence of altering work function (metal-contact) values on the DSSC performance parameters. All of the performance parameters (PCE, FF, Jsc, and Voc) are being improved when the value of the work function grows due to effective charge carrier transport and collection at back-metal contact. The PCE increased dramatically from 11.75% to 14.61%. It can be concluded that devices with high metal work functions are necessary for better photovoltaic performance.

**Figure 13:**
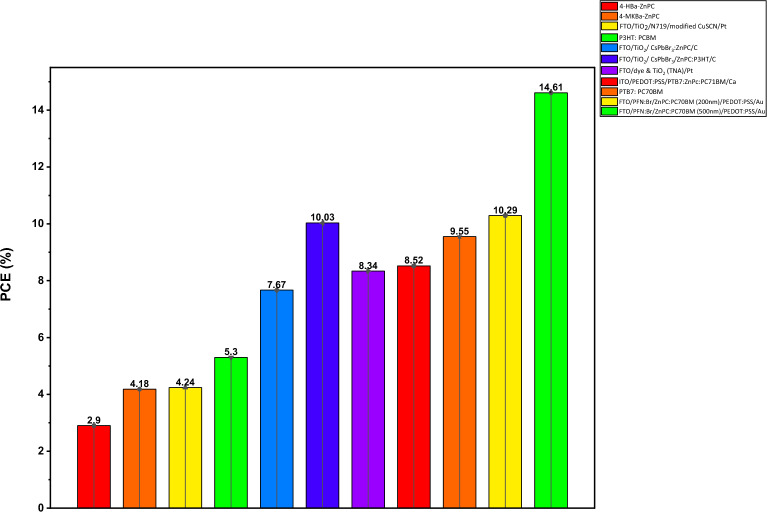
Sketch of comparison between simulated result with experimental results.

Table 3 compares the numerical analysis of the DSSCs with experimental data. The PCE of the simulated device model is better than experimental data from available research^65–71^. The difference between the theoretical and empirical results may be the consequence of reflection losses, the involvement of series and shunt resistances, and the effect of operating temperature. We discovered that correct parameters and layer configuration optimization can improve efficiency. However, PCE values greater than 8% have been observed for some dyes (see Fig. 13). The use of ZnPC:PC70BM as an absorber material with efficient electron and hole transport materials (i.e., PFN:Br and PEDOT:PSS) has improved the optoelectronic properties and might replace traditional dyes, which are concerned with stability and volatility issues for next-generation DSSCs. Consequently, this research also provides theoretical guidance for the actual usage of DSSC by improving its characteristics for the next-generation DSSC.

**Table 3:** Comparison of simulated result with experimental results.

Device configuration	PCE (%)	Refs
Experimental results		
4-HBa-ZnPc	2.99	^65^
4-MKBa-CoPc	4.18	^66^
FTO/TiO_2_/N719/CuSCN/C	4.24	^66^
P3HT:PCBM/ZnPC	5.3	^67^
FTO/TiO_2_/ CsPbBr_3_:ZnPc/C	7.67	^68^
FTO/TiO_2_/ CsPbBr_3_/ZnPc:P3HT/C	10.03	^68^
FTO/dye & TiO_2_ (TNA)/Pt	8.34	^69^
ITO/PEDOT:PSS/PTB7:ZnPc:PC71BM/Ca	8.52	^70^
PTB7:PC70BM	9.55	^71^
Simulation results		
FTO/PFN:Br/ZnPC:PC70BM (200 nm)/PEDOT:PSS/Au	10.29	This study
FTO/PFN:Br/ZnPC:PC70BM (500 nm)/PEDOT:PSS/Au	14.61	This study

## Conclusions

This work has offered valuable insights into how the characteristics of DSSCs might be altered during the commercial manufacture of solar cells. The SCAPS-1D software package is utilized to optimize and design the desired DSSC structure of FTO/PFN:Br/ZnPC:PC70BM/PEDOT:PSS/Au. The cell's performance was maximized by making suitable modifications to thickness, series and shunt resistances, metal-contact work functions, carrier mobility, and trap density. The analysis shows that the material's photovoltaic properties have improved by reducing the defect density, adjusting the absorber layer thickness appropriately, and raising the charge carrier mobility. According to our findings, the best value for thickness is 500 nm, defect density is 1 × 10^12^ cm^−3^ and carrier mobility is 5 cm^2^/Vs to achieve high productivity. By optimizing all the prime parameters, we could construct DSSC with a high efficiency of 14.61% and the simulated result suggests that the performance of DSSC devices will increase in the near future. Consequently, solar energy can potentially increase energy security, provided that the conditions of its production are correctly managed. Numerical simulation of solar cell designs provides companies with essential information while saving time and money. To support the theoretical work described in this research, an experimental approach to the performance of model DSSC solar cells is suggested.

## Data Availability

The authors declare that the data supporting the findings of this study are available within the article.

## References

[1] Gong J, Sumathy K, Qiao Q, Zhou Z (2017). Review on dye-sensitized solar cells (DSSCs): Advanced techniques and research trends. Renew. Sustain. Energy Rev..

[2] O'regan B, Grätzel MA (1991). low-cost, high-efficiency solar cell based on dye-sensitized colloidal TiO_2_ films. Nature.

[3] Grätzel M (2003). Dye-sensitized solar cells. J. Photochem. Photobiol., C.

[4] Sharma K, Sharma V, Sharma SS (2018). Dye-sensitized solar cells: fundamentals and current status. Nanoscale Res. Lett..

[5] Iqbal MA, Malik M, Shahid W, Din SZU, Anwar N, Ikram M, Idrees F (2022). Materials for photovoltaics: Overview, generations, recent advancements and future prospects. Thin Films Photovoltaics.

[6] Gregg BA, Hanna MC (2003). Comparing organic to inorganic photovoltaic cells: Theory, experiment, and simulation. J. Appl. Phys..

[7] Wang J, Liu K, Ma L, Zhan X (2002). Triarylamine: Versatile platform for organic, dye-sensitized, and perovskite solar cells. Chem. Rev..

[8] Khan M, Iqbal MA, Malik M, Hashmi SUM, Bakhsh S, Sohail M (2023). Improving the efficiency of dye-sensitized solar cells based on rare-earth metal modified bismuth ferrites. Sci. Rep..

[9] Jahantigh F, Ghorashi SB, Belverdi AR (2018). A first principle study of benzimidazobenzophenanthrolin and tetraphenyldibenzoperiflanthene to design and construct novel organic solar cells. Physica B: Condens. Matter.

[10] Abdelaziz S, Zekry A, Shaker A, Abouelatta M (2020). Investigating the performance of formamidinium tin-based perovskite solar cell by SCAPS device simulation. Opt. Mater..

[11] Lin L, Jiang L, Li P, Xiong H, Kang Z, Fan B, Qiu Y (2020). Simulated development and optimized performance of CsPbI_3_ based all-inorganic perovskite solar cells. Sol. Energy.

[12] Biplab SRI, Ali MH, Moon MMA, Pervez MF, Rahman MF, Hossain J (2020). Performance enhancement of CIGS-based solar cells by incorporating an ultrathin BaSi_2_ BSF layer. J. Comput. Electron..

[13] Bauer A, Sharbati S, Powalla M (2017). Systematic survey of suitable buffer and high resistive window layer materials in CuIn_1−x_Ga_x_Se_2_ solar cells by numerical simulations. Sol. Energy Mater. Sol. Cells.

[14] Huang CH, Chuang WJ (2015). Dependence of performance parameters of CdTe solar cells on semiconductor properties studied by using SCAPS-1D. Vacuum.

[15] Nowsherwan GA, Samad A, Iqbal MA, Mushtaq T, Hussain A, Malik M (2022). Performance analysis and optimization of a PBDB-T: ITIC based organic solar cell using graphene oxide as the hole transport layer. Nanomaterials.

[16] Korir BK, Kibet JK, Ngari SM (2021). Simulated performance of a novel solid-state dye-sensitized solar cell based on phenyl-C 61-butyric acid methyl ester (PC 61 BM) electron transport layer. Opt. Quant. Electron..

[17] Jahantigh F, Safikhani MJ (2019). The effect of HTM on the performance of solid-state dye-sanitized solar cells (SDSSCs): a SCAPS-1D simulation study. Appl. Phys. A.

[18] Noorasid NS, Arith F, Firhat AY, Mustafa AN, Shah AS (2022). SCAPS numerical analysis of solid-state dye-sensitized solar cell utilizing copper (I) iodide as hole transport layer. Eng. J..

[19] Ojotu GK, Babaji G (2020). Simulation of an optimized poly 3-hexylthiophene (P3HT) based solid state dye sensitized solar cell (ss-DSSC) using SCAPS. Int. J. Mod. Res. Eng. Technol..

[20] Nithya KS, Sudheer KS (2020). Device modelling of non-fullerene organic solar cell with inorganic CuI hole transport layer using SCAPS 1-D. Optik.

[21] Abdelaziz W, Shaker A, Abouelatta M, Zekry A (2019). Possible efficiency boosting of non-fullerene acceptor solar cell using device simulation. Opt. Mater..

[22] Sharma B, Mathur AS, Rajput VK, Singh IK, Singh BP (2022). Device modeling of non-fullerene organic solar cell by incorporating CuSCN as a hole transport layer using SCAPS. Optik..

[23] Wang M, Grätzel C, Zakeeruddin SM, Grätzel M (2012). Recent developments in redox electrolytes for dye-sensitized solar cells. Energy Environ. Sci..

[24] Suzuki K, Yamaguchi M, Kumagai M, Yanagida S (2003). Application of carbon nanotubes to counter electrodes of dye-sensitized solar cells. Chem. Lett..

[25] Yum JH, Chen P, Grätzel M, Nazeeruddin MK (2008). Recent developments in solid-state dye-sensitized solar cells. ChemSusChem Chem. Sustain. Energy Mater..

[26] Manfredi N, Bianchi A, Causin V, Ruffo R, Simonutti R, Abbotto A (2014). Electrolytes for quasi solid-state dye-sensitized solar cells based on block copolymers. J. Polym. Sci., Part A: Polym. Chem..

[27] Syafiq U, Ataollahi N, Scardi P (2020). Progress in CZTS as hole transport layer in perovskite solar cell. Sol. Energy.

[28] Calió L, Kazim S, Grätzel M, Ahmad S (2016). Hole-transport materials for perovskite solar cells. Angew. Chem. Int. Ed..

[29] Shum K, Chen Z, Qureshi J, Yu C, Wang JJ, Pfenninger W, Vockic N (2010). Synthesis and characterization of CsSnI_3_ thin films. Appl. Phys. Lett..

[30] Lancelle-Beltran E, Prené P, Boscher C, Belleville P, Buvat P, Lambert S (2008). Solid-state organic/inorganic hybrid solar cells based on poly (octylthiophene) and dye-sensitized nanobrookite and nanoanatase TiO_2_ electrodes. Eur. J. Inorg. Chem..

[31] Sun S, Salim T, Mathews N, Duchamp M, Boothroyd C, Xing G (2014). The origin of high efficiency in low-temperature solution-processable bilayer organometal halide hybrid solar cells. Energy Environ. Sci..

[32] Yue G, Wu J, Xiao Y, Ye H, Lin J, Huang M (2011). Flexible dye-sensitized solar cell based on PCBM/P3HT heterojunction. Chin. Sci. Bull..

[33] Ahmadi M, Asemi M, Ghanaatshoar M (2018). Mg and N co-doped CuCrO_2_: A record breaking p-type TCO. Appl. Phys. Lett..

[34] Yella A, Lee HW, Tsao HN, Yi C, Chandiran AK, Nazeeruddin MK (2011). Porphyrin-sensitized solar cells with cobalt (II/III)–based redox electrolyte exceed 12 percent efficiency. Science.

[35] Burschka J, Pellet N, Moon SJ, Humphry-Baker R, Gao P, Nazeeruddin MK (2013). Sequential deposition as a route to high-performance perovskite-sensitized solar cells. Nature.

[36] Iqbal MA, Anwar N, Malik M, Al-Bahrani M, Islam M, Choi J (2023). Nanostructures/graphene/silicon junction-based high-performance photodetection systems: progress, challenges, and future trends. Adv. Mater. Interfaces..

[37] Mathew S, Yella A, Gao P, Humphry-Baker R, Curchod BF, Ashari-Astani N, Tavernelli I (2014). Dye-sensitized solar cells with 13% efficiency achieved through the molecular engineering of porphyrin sensitizers. Nature Chem..

[38] Burgelman M, Nollet P, Degrave S (2000). Modelling polycrystalline semiconductor solar cells. Thin Solid Films.

[39] Liu F, Zhu J, Wei J, Li Y, Lv M, Yang S (2014). Numerical simulation: toward the design of high-efficiency planar perovskite solar cells. Appl. Phys. Lett..

[40] Rokesh K, Pandikumar A, Jothivenkatachalam K (2014). Dye sensitized solar cell: a summary. Mater. Sci. Forum.

[41] Sharma K, Sharma V, Sharma SS (2018). Dye-sensitized solar cells: fundamentals and current status. Nanoscale Res. Lett..

[42] Shah SA, Guo Z, Sayyad MH, Sun J (2021). Optimizing zinc oxide nanorods based DSSC employing different growth conditions and SnO coating. J. Mater. Sci.: Mater. Electron..

[43] Shah SA, Guo Z, Sayyad MH, Abdulkarim S (2021). Layer-by-layer titanium (IV) chloride treatment of TiO_2_ films to improve solar energy harvesting in dye-sensitized solar cells. J. Electron. Mater..

[44] Shah SA, Sayyad MH, Nasr N, Toor RA, Sajjad S, Elbohy H, Qiao Q (2017). Photovoltaic performance and impedance spectroscopy of a purely organic dye and most common metallic dye based dye-sensitized solar cells. J. Mater. Sci.: Mater. Electron..

[45] Nithya KS, Sudheer KS (2020). Numerical modelling of non-fullerene organic solar cell with high dielectric constant ITIC-OE acceptor. J. Phys. Commun..

[46] Patel MJ, Gupta SK, Gajjar PN (2020). Electronic structure and optical properties of β-CuSCN: A DFT study. Mater. Today: Proc..

[47] Hu R, Zhang W, Xiao Z, Zhang J, Su X, Wang G (2021). Charge photogeneration and recombination in ternary polymer solar cells based on compatible acceptors. J. Mater. Sci..

[48] Socol M, Preda N, Petre G, Costas A, Rasoga O, Popescu-Pelin G (2020). MAPLE deposition of binary and ternary organic bulk heterojunctions based on zinc phthalocyanine. Coatings.

[49] Hossain MI, Alharbi FH, Tabet N (2015). Copper oxide as inorganic hole transport material for lead halide perovskite based solar cells. Sol. Energy..

[50] Siddique SA, Arshad M, Naveed S, Mehboob MY, Adnan M, Hussain R (2021). Efficient tuning of zinc phthalocyanine-based dyes for dye-sensitized solar cells: a detailed DFT study. RSC Adv..

[51] Tan K, Lin P, Wang G, Liu Y, Xu Z, Lin Y (2016). Controllable design of solid-state perovskite solar cells by SCAPS device simulation. Solid-State Electron..

[52] Stoumpos CC, Malliakas CD, Kanatzidis MG (2013). Semiconducting tin and lead iodide perovskites with organic cations: phase transitions, high mobilities, and near-infrared photoluminescent properties. Inorg. Chem..

[53] Hipps KW, Mazur U (2012). Electron affinity states of metal supported phthalocyanines measured by tunneling spectroscopy. J. Porphyrins Phthalocyanines.

[54] Seo JH, Nguyen TQ (2008). Electronic properties of conjugated polyelectrolyte thin films. J. Am. Chem. Soc..

[55] Chen CW, Hsiao SY, Chen CY, Kang HW, Huang ZY, Lin HW (2015). Optical properties of organometal halide perovskite thin films and general device structure design rules for perovskite single and tandem solar cells. J. Mater. Chem. A.

[56] Aboura FB, Duché D, Simon JJ, Escoubas L (2015). Ellipsometric study of the optical transitions of PC60BM and PC70BM thin films. Chem. Phys..

[57] Sahoo D, Manik NB (2023). Study on the effect of temperature on electrical and photovoltaic parameters of lead-free tin-based Perovskite solar cell. Indian J. Phys..

[58] McIntosh KR, Honsberg CB. The influence of edge recombination on a solar cell's IV curve. InProc. 16th PVSEC, Glasgow 2000 May (pp. 1651–1654).

[59] Tvingstedt K, Gil-Escrig L, Momblona C, Rieder P, Kiermasch D, Sessolo M (2017). Removing leakage and surface recombination in planar perovskite solar cells. ACS Energy Lett..

[60] Singh R, Sandhu S, Lee JJ (2019). Elucidating the effect of shunt losses on the performance of mesoporous perovskite solar cells. Sol. Energy.

[61] Karthick S, Velumani S, Bouclé J (2020). Experimental and SCAPS simulated formamidinium perovskite solar cells: A comparison of device performance. Sol. Energy.

[62] Anwar F, Afrin S, Satter SS, Mahbub R, Ullah SM (2017). Simulation and performance study of nanowire CdS/CdTe solar cell. Int. J. Renew. Energy Res.

[63] Derry GN, Kern ME, Worth EH (2015). Recommended values of clean metal surface work functions. J. Vacuum Sci. Technol. A: Vacuum Surfaces Films.

[64] Thahab, S., M., Hassan, H., A., & Hassan, Z. Effects of metal work function and operating temperatures on the electrical properties of contacts to n-type GaN. IEEE International Conference on Semiconductor Electronic. 816–819 (2006).

[65] Sevim AM, Çakar S, Özacar M, Gül A (2018). Electrochemical and photovoltaic properties of highly efficient solar cells with cobalt/zinc phthalocyanine sensitizers. Sol. Energy.

[66] Dematage, N. Dye-sensitized and Semiconductor-sensitized Solid State Solar Cells Utilizing CuSCN and CuI as Hole Conducting Materials (Doctoral dissertation, 静岡大). (2014).

[67] Kadem B, Hassan A, Göksel M, Basova T, Şenocak A, Demirbaş E (2016). High performance ternary solar cells based on P3HT: PCBM and ZnPc-hybrids. RSC Adv..

[68] Duan J, Xu H, Sha WEI, Zhao Y, Wang Y, Yang X (2019). Inorganic perovskite solar cells: An emerging member of the photovoltaic community. J. Mater. Chem. A.

[69] Ge Z, Wang C, Chen Z, Wang T, Chen T, Shi R (2021). Investigation of the TiO_2_ nanoparticles aggregation with high light harvesting for high-efficiency dye-sensitized solar cell. Mater. Res. Bull..

[70] Stylianakis MM, Konios D, Viskadouros G, Vernardou D, Katsarakis N, Koudoumas E (2017). Ternary organic solar cells incorporating zinc phthalocyanine with improved performance exceeding 8.5%. Dyes Pigments.

[71] Zheng Y, Wang G, Huang D, Kong J, Goh T, Huang W (2018). Binary solvent additives treatment boosts the efficiency of PTB7: PCBM polymer solar cells to over 9.5%. Solar Rrl..

